# Quality-of-Life Assessments in Zoo Animals: Not Just for the Aged and Charismatic

**DOI:** 10.3390/ani13213394

**Published:** 2023-11-01

**Authors:** Michelle Campbell-Ward

**Affiliations:** 1 Sydney School of Veterinary Science, Faculty of Science, The University of Sydney, Camden, NSW 2570, Australia; michelle.campbell@sydney.edu.au; 2 School of Agricultural, Environmental and Veterinary Sciences, Charles Sturt University, Wagga Wagga, NSW 2650, Australia

**Keywords:** quality of life, assessment, zoo animal, geriatric, euthanasia, decision-making, animal welfare

## Abstract

**Simple Summary:**

Zoos should aim to provide all of their animals with a good quality of life throughout life. Although quality of life is a complex notion that is difficult to define and measure, some modern zoos and zoo industry associations have started to develop and implement formalized quality-of-life assessment tools for this purpose. These tools have been used predominantly to assess the health and welfare of geriatric zoo mammals to assist in decision-making in relation to end-of-life care, including the timing of euthanasia. There is scope to improve the accuracy of these tools and to extend their use to assess the quality of life of non-mammalian species (e.g., birds and reptiles) and animals at other life stages (e.g., young adults) to enhance animal welfare. This review summarizes what quality of life is, why and how we should assess it in zoo animals, and what challenges this poses. It also identifies some learnings from quality-of-life assessments in humans and domestic species that can be applied to zoo animals and suggests directions for future innovations in this field.

**Abstract:**

Zoos should aim to provide all of their animals with a good quality of life (QoL) throughout all life stages. In parallel with the evolution of QoL assessment questionnaires and tools in human and domestic animal settings, in recent times, some individual zoos and zoo industry associations have incorporated such instruments into their animal management practices. This has been conducted predominantly to inform, monitor, and document end-of-life decision-making for large, charismatic mammals. There is scope to expand the use of these tools to improve their utility, validity, reliability, and value to an animal welfare program. Assessment of QoL is a complex task given that the notion being measured is abstract and self-determined, and the design and purpose of the tools to do this require careful consideration. This review explores the QoL concept as it applies to animals, the assessment indications and methodologies relevant to a zoo setting, and the importance of considering QoL at any life stage across species. An overview of current thinking and the applications and limitations of QoL evaluation of captive wild animals is offered to promote and aid facility practice reviews and to help direct future innovations that leverage concurrent and converging advances in zoo animal welfare science.

## 1. Introduction

The exponential growth of the development and use of quality-of-life (QoL) measures for people and animals in recent decades has been driven by a strongly perceived need for such tools [1]. Despite unity regarding its importance, the conceptualization of QoL has proven to be contentious, and as a result, a wide variety of approaches to and methods for its evaluation have been and are still being explored [2,3]. In zoos, the early evolution and adoption of animal QoL assessments is recognized as an important step in the advancement of the care and welfare of aged animals [4], with applications to date focused predominantly on larger, and arguably more charismatic, mammals, as an aid in end-of-life decision-making. This has been a welcome and useful innovation, and global accreditation bodies are increasingly recommending their members adopt such tools for animal management [5,6,7]. However, the process remains largely ad hoc, limited in its application and lacks validated benchmarks for most species. There is widespread agreement that effective evaluation of the QoL of zoo animals to detect and minimize QoL threats and maximize meaningful positive experiences is needed. There is less clarity on exactly how to do that.

This review explores the QoL concept as it applies to animals, the assessment indications and methodologies relevant to zoo and aquarium species, and the value of considering QoL at any life stage for any species. The objectives are to promote and aid facility practice reviews and to help shape the next stages in the development of QoL evaluation for captive wild animals that leverage concurrent and converging advances in zoo animal welfare science.

## 2. Quality of Life—What Is It?

The term ‘quality of life’ (QoL) is used frequently in society and animal management settings, but the concept is abstract, broad, and complex, and it remains variably defined, even as applied to humans [8,9]. There are diverse and debated approaches to explaining QoL, with the incorporation of factors such as individual needs, expectations, phenomenological viewpoints, hedonism, flourishing, preferences, and life satisfaction [10,11]. Examples of definitions of QoL are provided in Table 1. In human settings, these include reference to a conscious cognitive judgment [12] and a personal perception of an individual’s life in relation to the culture and value systems in which they live [13]. Although many definitions of QoL focus on subjective judgements, some authors argue that objective factors should be included [14,15]. With respect to animal QoL, references to culture and values are absent [16], and some definitions are similar or identical to modern definitions of ‘animal welfare’ [17], which is itself a complex notion that lacks a universally agreed upon definition. There are definitions that erroneously include inputs presumed to affect the welfare and QoL of animals, as well as the individual’s response to those inputs, thereby confusing indicators of QoL with QoL itself [18]. Others limit the definition to the animal’s evaluation, perception, and affective response [19,20,21], and/or highlight the balance of positive and negative experiences [19,22].

Despite the lack of consensus on and precision of an operational definition of the term, the concept of QoL has evolved substantially as applied to both humans and animals in recent times, with attempts to clarify what it incorporates. Early uses of the term originated in health documentation and the medical literature and focused on functional capacity [26] as a tool for physicians. As a consequence, the terms QoL, health, and health-related QoL (HRQoL) started to be used interchangeably [27,28], and confusion about the difference between and overlap of these terms persists [9,16].

More recent descriptions of QoL usually encompass notions that extend beyond physical health to include the psychological state [16,29]. In people, the level of independence and personal beliefs are often also included [26]. For both people and animals, QoL is proposed by some to encompass social relationships and environmental interactions [25,30,31]. There is a sense that it is determined not only by an individual’s life circumstances and opportunities but also by what uniquely matters to them, what they like or prefer doing, and their ability to fulfil their interests and enjoy life [30,32]. This evolution in thinking has paralleled the transition of the concept of animal welfare from one focused on the abolition of negative conditions to one that emphasizes positive mental experiences in terms of satisfied needs and expectations [33]. It is also consistent with the framework that describes health as just one of the five domains of animal welfare and one of the four functional domains (alongside nutrition, environment, and behavioural interactions) that influence the affective state [34].

In an attempt to avoid creating a false distinction between animal welfare and QoL, Yeates [25] proposed that QoL applies over time. At any given moment, an animal has good, neutral, or bad welfare, but its QoL is a matter of its welfare during an extended period. Indeed, in this view, it follows that the terms ‘QoL’ and ‘animal welfare over time’ are synonymous. Similarly, Cussen and DiGangi [35] describe QoL as an individual animal’s affective state over time. The temporal component of welfare is often overlooked in traditional evaluations [36], whereas an assessment of QoL should consider all of the events that occur within a period. Furthermore, QoL is closely related to, and may be considered equivalent to, a number of other concepts, such as well-being (sometimes specified as subjective, emotional, psychological, or mental well-being), happiness, and contentment [37,38].

## 3. Quality-of-Life Assessments in Zoo Animals

### 3.1. Why Assess Quality of Life?

With their origins in human health care, QoL assessments have historically been conducted to aid decision-making for individual patients as a complement to clinical or laboratory tests [39]. This traditional HRQoL approach attempts to measure how much an illness or a disability affects an individual’s overall well-being and their ability to adapt to imposed changes [40]. The initial impetus behind developing the construct was to represent the patient’s interests in the decisions made about their medical care, reflecting the growing respect among healthcare professionals for patient autonomy and self-determination [3]. The process of evaluating HRQoL aims to minimize the risk of making incorrect assumptions about an individual’s QoL following a diagnosis, procedure, or treatment protocol [20]. QoL in human care settings is also measured in non-medical circumstances in which individuals are the responsibility of others, such as in care homes for the elderly [41]. Here, too, as in animal welfare, the focus is shifting from simply measuring the provision of basic care to include the experiences of individuals.

QoL measures can be used in clinical settings to prioritize problems, facilitate communication, screen for potential issues, identify preferences, monitor changes or treatment responses, and train new staff [42]. They can also be used in clinical audits and in clinical governance to promote both a systematic evaluation of outcomes and continual improvements [42]. Within research settings, QoL assessments are useful in evaluating health care interventions and treatments [43], defining potential endpoints in clinical trials [44], identifying health inequalities [45], understanding the burden of disease, and assisting in resource allocation [46]. Although these indications have mostly played out formally in human medicine, they are all potentially relevant to veterinary and animal care settings.

In a veterinary context, QoL assessments, or perhaps more specifically, HRQoL assessments, are not new [47]. Informal, mental, subjective assessments by veterinarians and animal caregivers have always facilitated decision-making processes, but historically, documentation of that evaluation may have been lacking, rudimentary, or inconsistent. The decisions informed by such assessments include if, when, and how to treat, and whether that treatment is intended to be curative or palliative, defining thresholds of declines relative to a subjectively determined point and the appropriateness of euthanasia in aged or critically unwell animals [20]. In recent times, there has been a move to develop more formalized QoL assessment tools for consistency and communication in assorted animal care settings [20], especially as treatment options increase in availability and affordability and animals in captive environments are living longer lives [48]. In zoos, the focus of the application of such tools has been on mammals approaching or exceeding their expected longevity [49,50,51].

The decisions surrounding when and how to end an unwell or aged animal’s life can be some of the most challenging in terms of balancing animal welfare considerations and human sensitivities [52]. Some people may be concerned that ending a life at a point in time deemed ‘too early’ might miss the opportunity to observe that the animal will indeed respond positively to a new or advanced treatment. This occurs even if evidence of efficacy is scant or absent [53]. There is tension created by the tendency to overestimate the potential benefits of continuing to live weighed up against the difficulties of foreseeing potential harms [52,54]. All decision-makers and the diversity of people affected by a pending end-of-life decision are influenced by their emotions as well as their training, experience, personal beliefs, and extraneous factors [55,56]. When there are many people involved, consensus on the optimal approach may, therefore, be challenging to achieve. Föllmi et al. [49] explained that the decision to perform euthanasia in geriatric zoo animals, mammals in particular, is usually a highly complex procedure involving ethical, medical, emotional, and sometimes political factors, which can delay a euthanasia decision to the detriment of the animal’s welfare. This is especially true for charismatic or so-called ‘cute’ animals, where the recognized empathy bias towards certain taxa [57] is upheld. Methods to minimize bias in the decision-making process are necessary to ensure interventions and their timing are appropriate to prevent unnecessary suffering and are understood by the heterogeneous group of stakeholders impacted. In their use in a zoo environment, objective QoL assessment tools and processes can help foster a culture of trust and collaboration, educate and prepare staff at all levels of an institution for an animal’s death, clarify expectations, promote compassionate animal care and welfare as key principles, and help explain euthanasia decisions [50,58].

Regardless of taxa, the precise timing of euthanasia should be based on appropriate clinical judgment, informed by signs that indicate an irreversible decline in welfare and QoL to a point approaching, at, or beyond that deemed inhumane. To assist this process, objective methods of assessing negative affective states, such as pain and distress, are invaluable and, where available, should be incorporated into decision-making tools [53].

If QoL is taken to be equivalent to welfare over time as discussed earlier, HRQoL assessments at any life stage permit a systematic consideration of whether and how a specific diagnosis will adversely affect the welfare of an individual animal, accounting for their known preferences, behaviours, and interests. These objective assessments can also be used to compare the predicted or observed impacts of different treatment options, including no treatment at all [59] for an animal that has yet to reach the point of irreversible decline, elderly or otherwise.

Beyond end-of-life veterinary care, QoL assessments conducted on animals at other life stages can have much wider applications: they can inform husbandry improvements and other management factors; assess the impact of enclosure design, enclosure moves, introductions, and enrichment programs; aid in collection planning; and help enable the projection of future harms or benefits to an animal related to an anticipated change or a lack of a change [60,61]. A dominant contemporary notion is that QoL and animal welfare apply at the individual level [1]. However, QoL assessments conducted at a group or species level, which may take the form of group animal welfare assessment [47], may also help inform minimum and best practice standards, as demonstrated for dog rehoming centres [62].

QoL assessments in zoos may also have benefits for staff beyond their use in decision-making and communication. Research has demonstrated that zoo personnel rank making improvements in animal QoL a key motivator of their work [63]. Similarly, others have found that a management focus on the psychological welfare of zoo animals to fill perceived gaps may lead to greater zookeeper job satisfaction [64]. Keeper involvement in the development, adoption, and regular critical review of QoL tools is a way of empowering such staff to meaningfully evaluate the outcomes of their work while fostering a culture of professional growth.

### 3.2. Observer-Reported Outcome Measures

QoL can only truly be measured from the individual’s perspective [1]. As such, the self-report (a subjective judgement expressed by a person) is the gold standard for QoL assessment in humans [26]. In those unable to self-report, such as very young children or those with cognitive impairment, alternatives such as the use of an observer or proxy (e.g., a parent or other family member, physician, or nurse) is required to assess QoL on the individual’s behalf. This is referred to as an observer-reported outcome (ORO) measure [46,65]. As animals cannot directly express how they feel to humans, all animal QoL assessment methods and tools are classified as OROs [32]. Therefore, it is the responsibility of the veterinarians, animal caregivers, and managers to estimate an animal’s QoL based on criteria deemed meaningful to that species and the individual animal [55], and it is important that assessments be made by observers familiar with both [66].

The limitations of this process must be acknowledged. Proxy assessments are not always accurate [65,67]. McCusker and Stoddard [68] compared responses to a general QoL measure of close relatives who were acting as proxies with those of seriously ill people. They found that proxies consistently underestimated the ill patient’s QoL if the condition was deemed terminal or if the caretaker did not live in the same household as the patient. QoL reports provided by parents of medically unwell children are influenced by their own well-being and do not match those provided by the children themselves [69]. Additionally, proxy factors such as gender and career experience can influence responses to QoL evaluations of animals. When male and female owners were presented with a questionnaire regarding their own dog’s perceived levels of stress, male participants ranked the stress levels as lower than the ranking provided by female participants [70]. In geriatric zoo animals, QoL ratings assessed by veterinarians demonstrated interobserver differences related to career experience and area of specialty, with those categorized as zoo veterinarians and pathologists scoring QoL higher than interns, surgeons, and anaesthetists evaluating the same animal [49]. Consequently, there is a risk that OROs may never accurately reflect an individual animal’s life experience [71] and cannot be realistically expected to capture all aspects of life that are important to an individual [42].

These limitations should not discourage zoo professionals from designing and evaluating QoL assessment tools. Rather, understanding them should inspire a significant effort in working towards developing robust, validated techniques with regular critical review.

### 3.3. Quality-of-Life Scaling to Reflect Positive-Negative Balance

**Figure 1:**
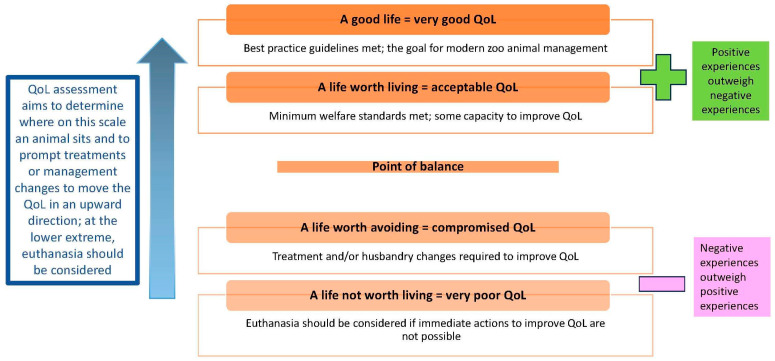
A simple four-point quality-of-life (QoL) scale for zoos extending from a good life at the extreme end of the positive valence to a life not worth living at the other extreme, acknowledging the continuum that exists between each point (adapted from Green and Mellor [33]).

Although QoL is multidimensional and assessments vary in their complexity and granularity of components, the ultimate goal of an individual QoL assessment is to determine if QoL is good or bad, and therefore, what, if any, action should be taken. To this end, QoL can be helpfully and practically contextualized on a simple four-point QoL scale [33], acknowledging the continuum that exists between each point. There are two points above a point of balance, defined as the point where salient positive and negative experiences are equally balanced, and two points below. An animal may be considered to have a good life or a life worth living in the positive valence, and a life worth avoiding or a life not worth living on the negative end of the scale (Figure 1). Browning [72] supports the bidirectional nature of this type of scale but suggests that the total possible intensity on either side of the point of balance is not necessarily equal.

Zoos should aim to provide all of their animals with a good life throughout all life stages. Not only must there be full compliance with all legally mandated minimum standards or codes of practice but also with best practice guidelines to meet increasing community and industry expectations [73,74].

The life of an animal deemed not worth living should be improved rapidly by veterinary treatment and/or a change in husbandry practices. Failing that, euthanasia is the only humane option.

The challenge for zoo practitioners and researchers then is to develop QoL assessment tools that determine with an acceptable level of precision where an animal perceives its position on this scale: an inordinately difficult and complex undertaking. Despite decades of extensive research and substantial funding, a uniformly applied best approach suitable for all contexts has not been identified for QoL evaluation in people [3]. It is, therefore, reasonable to expect that a suite of options will be necessary to accommodate the diversity of species and QoL applications in zoos. Although veterinary QoL instruments for animals have been most commonly developed for companion animals [16], systematic reviews have revealed that there are limited numbers of published dog and cat QoL assessment tools and that among those that do exist, there is a low level of validation and consistency [20,75,76]. Nevertheless, given the importance of the QoL concept, using, adapting, and continually refining the tools that are in development is a preferred approach to not trying at all [38].

### 3.4. Types of Quality-of-Life Assessments Relevant to Zoos

While research and development of animal QoL tools are in their infancy, there is value in exploring the different types of QoL assessments that may be useful in a zoo setting. Ultimately, the type of assessment conducted will depend on the reason for its use.

Firstly, a distinction can be made between generic and disease- or condition-specific instruments [20]. Generic tools intend to measure all dimensions of QoL and can, therefore, be applied to healthy individuals as well as those with recognized health or other issues. The advantage of generic tools is that they provide a holistic overview of QoL and allow for comparisons between specific situations [77]. These can be thought of as equivalent to comprehensive animal welfare assessments that are described in detail elsewhere [17,34,36,74]. Although they are broad in their application, it is recommended that such generic assessments include a mechanism to account for individual differences to improve their accuracy in estimating QoL [16]. For example, an animal’s early life history or physical capacity to perform natural behaviours could influence how an assessment, or a sub-component of it, is interpreted.

A disease-specific QoL assessment includes measurements of dimensions or parameters specific to a certain diagnosis and is more likely to be sensitive to changes in the clinical condition or responses to treatments [78]. For example, gait analysis is relevant to monitoring lameness and may be incorporated into a degenerative joint disease assessment for a particularly susceptible species. Disease-specific assessments can also be used in a forward-looking manner to systematically consider the relative impact of potential stressors and other QoL risks associated with interventions deemed necessary or recommended to treat a given condition. Some medical treatments or interventions are expected to have an immediate positive benefit on QoL (e.g., relief of pain with effective and easy-to-administer analgesia). Others are likely to have a negative impact in the short term (e.g., due to hospitalization, surgery, confinement, altered social settings, or unpleasant medications) but have the potential to improve welfare in the long term. The management of this balance may vary not only with time but also with species, individual, and condition, and will benefit from ongoing proactive review.

In a zoo setting, a basic generic template tool with customized inclusions for different species or taxonomic groups is recommended, and it may be appropriate to have subsets of indicators relevant to a particular age or other demographic cohorts. Disease-specific assessments may be appropriate for welfare-compromising conditions that are common in particular species or groups of species held in zoos and aquariums. It is possible to develop a tool that combines elements of both generic and disease-specific assessments, and these may prove to be optimal [79], especially given that animals may present with multiple comorbidities.

Geriatric QoL assessments in zoos have typically taken the form of generic assessments. As they are only initiated when animals reach a particular age [50], interpretation of the results is difficult when there is no baseline for the species or individual for comparison. Facilities are encouraged to work on establishing reference guidelines for any bespoke tools that are used, and it is important that they incorporate evidence-based criteria on common QoL-relevant age-related concerns in the species or taxonomic group of concern.

### 3.5. Assessment Methods

The most basic method of assessing animal QoL involves asking a proxy what they feel the QoL is on a Likert scale. This may use the scale described in Figure 1, a differently worded scale, or an equivalent numerical scale. Alternatively, a proxy may be asked to describe the QoL in one word. In either case, these so-called ‘human intuitive judgements’ should be treated with caution [72] because although they are quick to perform, they are highly subjective and prone to various personal biases.

One approach to overcome the crude simplicity of these methods is to use multiple assessors and calculate the range or average as the result. This technique was utilized in a study evaluating the outcomes of phacoemulsification cataract surgery in 21 penguins [80]. A total of 20 staff members (7 avian management team members and 13 aviculturists) subjectively evaluated the QoL of the penguins, and their scores were averaged. Using a 4-point Likert scale with assigned quantitative values of poor (1), fair (2), good (3), and excellent (4), staff members were asked to subjectively evaluate each bird’s QoL before and after surgery. Keepers were also asked to describe any specific post-procedure behavioural changes observed for each penguin. The results revealed that 81% of the penguins were deemed to have a subjective improvement in QoL post-operatively based on the average of all assessors’ scores. This was consistent with the positive visual outcomes and improved mobility and behaviour (activity level, interactions, and behaviour responses) within their enclosure, but the degree of agreement between assessors was not reported.

In reality, acknowledging that QoL is a highly complex construct [1], it is unlikely that these kinds of over-simplified, unstructured methods, even with the use of multiple assessors, can ever be validated [76]. They risk missing subtle differences between patients and fail to provide the degree of evidence necessary to contribute meaningfully to welfare-enhancing decision-making. 

To address the significant limitations and caveats of using a single overall subjective measure, more comprehensive and multifactorial approaches have been developed. As discussed earlier, these have, to date, been largely focused on end-of-life decision-making in geriatric zoo mammals. One described format uses regular, systematic, but simple behavioural observations of an animal to infer its psychological well-being [81]. Zoo-housed elderly polar bears have been evaluated in this manner, where behaviours that occur in the absence of caretaker interactions and those that are caretaker-driven (e.g., interactive feedings, enrichment schedules, and training sessions) are assessed in combination [81]. Another described approach, commonly used in companion animal settings, uses a set of questions or items, of a variable number, designed for animal carers and/or veterinarians to answer [18]. The items included may be in the form of broad categories like appetite, or alternatively, asked as more targeted questions like “How much does the animal enjoy food?” [20]. In structured versions of such questionnaires the responses are typically converted into scores [76]. The EAZA Best Practice Guidelines for Elephants [6], for example, requires an assessor to provide a score from 0–4 for a number of categories of equal weight, including pain, medical conditions, appetite, physical condition, psychological health, social well-being, and mobility, with the option to include other issues not covered by the standard template. An evaluation guide is provided detailing how to interpret the sum of the category scores and what actions might be appropriate (monitoring, implementing accommodations, revised husbandry and treatment options, aggressive treatment, or euthanasia) depending on whether the QoL is deemed good, somewhat affected, significantly diminished, severely compromised, or very poor.

Föllmi et al. [49] described a less species-specific scoring system with a similar aim, i.e., to evaluate the physical condition and QoL of 70 geriatric zoo mammals. They found that although some component signs of the scoring system, such as lameness or vomiting, were easy to detect, quantify and/or qualify, others were less straightforward. The term ‘pain when standing up’, for example, was used when a strenuous effort was necessary for the animal to get to its feet; this observation may, however, vary depending on interpretations of what constitutes pain. Their system includes separate ratings (on a 1–10 scale) for pain, discomfort, and QoL, which together give a sub-score out of 30 that contributes to the total evaluation alongside sub-scores for symptoms, radiographic findings, and response to treatment. Definitions of pain, discomfort, and QoL are not provided, but it seems likely that many users, though perhaps not all, would interpret these as dependent variables.

Introducing the concept of weighting of selected indicators, Vogelnest and Talbot [50] described a QoL assessment checklist developed for individual geriatric zoo animals that includes behavioural characteristics, physical signs, and clinicopathological findings. Vinette-Herrin et al. [51] performed aged animal assessments with this tool on 90 animals from a variety of taxa, with just over one-third of the cohort having 1–4 follow-up serial assessments at 6–12 month intervals. Four individuals were euthanized as a direct result of the initial assessment. Another 24 were euthanized based on follow-up assessments or predetermined decisions to euthanize if conditions deteriorated. Approximately 50% of total cases remained stable, while nine cases had improved scores following the implementation of individualized care plans developed in response to early recognition of disease and age-related changes. The favourable fate of this latter group demonstrates the value of performing these assessments before significant and overt declines have occurred.

With the type of zoo-based assessments described above and others that have been developed for primates in laboratory settings [82], it is common practice to assemble a team of people to collaboratively complete the assessment and/or interpret the findings. A team approach ensures that multiple perspectives are accounted for, with participants meaningfully contributing to the decision-making dataset in the areas that encompass their expertise [82]. The people involved will vary with the institutional structure and size but ideally include care staff who are familiar with the individual, a veterinarian, a welfare officer or advisor, animal or institutional managers or curators, and a behavioural biologist or similar with a good understanding of species-specific norms. Some zoos elect to create a formalized committee to oversee such welfare-related processes, while others create temporary teams on a case-by-case or taxonomically-relevant basis.

The adoption of a multi-indicator aggregated approach to assess QoL is supported by welfare science and in a zoo setting has the advantage of being potentially useful across a broad range of species and contexts [72]. As discussed earlier, a well-designed generic QoL survey or checklist is equivalent to an animal welfare assessment, and the terms can be used interchangeably. In this regard, the purpose of QoL assessments need not be restricted to the evaluation of geriatric animals and end-of-life decision-making. The precise composition of an assessment protocol and how heavily each indicator contributes to the cumulative result will be context-dependent. The time available, type of operation, and reason for the assessment can determine the format. Scientific robustness relies on species-relevant parameters with high levels of inter-observer reliability, trained assessors, and ideally, measures that have been validated against other known welfare-relevant indicators. A balance must be struck between cost-effective feasibility, impositions on the animals and caretakers, and meaningful results. QoL checklists should generally not require access to scientific assays or specialized equipment but can reference the medical and keeping records. It is important to incorporate indicators that will realistically and objectively assess the animal’s QoL, are practical to measure within the constraints of the zoo environment [61], and can be applied to a variety of species within a taxonomic group.

A number of individual zoo animal- and species-specific welfare assessment tools and frameworks have been developed as aids in animal welfare management, including online versions and some that incorporate visual tracking [60,74]. The Animal Welfare Assessment Grid (AWAG), for example, has been successfully applied to zoo-housed primates, large felids, giraffes, and scimitar-horned oryx [61]. It is recommended that these tools and frameworks be used as the basis for further development of generic zoo animal QoL assessments, especially those for non-geriatric life stages and non-mammalian species. The results of a critical review of prevailing equine welfare assessment tools as candidates for QoL evaluation of chronically ill horses [21] provides some guidance and caution. The authors found that the most suitable instruments are restricted to those that prioritise the subjective mental experience of the animal, those that integrate criteria into one overall grade, and those that focus on long-term as opposed to momentary states. Some adaptations tailored to suit the context may be required.

### 3.6. Selecting Indicators for a Quality-of-Life Assessment

The challenge in developing a QoL assessment questionnaire or checklist is identifying and selecting objective, reliable, valid, and largely quantitative indicators that collectively can measure or at least reveal empiric but indirect information about a state that is subjective, self-determined, variable over time, and influenced by individual and species-specific factors [61,83]. Animals are complex organisms, and the factors that contribute to their QoL are numerous. Welfare assessment techniques that were initially developed and later refined in the agricultural sector [84] with subsequent spill over into research settings and other animal industries provide a good baseline. 

Even in well-studied species such as domestic cattle, pigs, and poultry, there is no single universal measure that accurately describes an individual’s welfare [72]. Indicators may be systematically evaluated by direct observation of the animals and their environments and/or by reviewing available records [85]. They may be measured in a binary fashion (e.g., presence/absence), via a scoring system or simply quantified [86], and recorded at a single point in time [87] or, as is more appropriate for QoL assessment, tracked longitudinally [88]. Important challenges and limitations of using indicators from domestic animal welfare science to accurately evaluate the welfare of zoo animals include the wide variety of species, their relative unfamiliarity to some observers, and the limited research on the needs and husbandry of some taxa [89]. Additionally, if handling is required to measure a particular indicator, this may pose risks to the animal and/or handler and adversely affect the results. 

There are two broad categories of indicators: resource-based measures and output-based measures [90]. Resource-based measures attempt to infer the welfare status of animals from the inputs and logistical practices that go into caring for them, such as the quality of substrate, stocking density, ventilation, feed trough size, food provided, lighting, hygiene, and staff training. Such parameters are often easy to define and measure and are repeatable between observers. However, they lack an animal-centric perspective, and their relationship to animal welfare is debated [91]. The provision of preferred and motivating resources and an appropriate level of environmental complexity are vital, but individual differences may exist, and keeper ratings of preference do not always correlate with the animal’s actual preference [92]. Conversely, output-based measures aim to gather more direct evidence regarding the welfare state of an animal. As they relate to zoo animals, these may be divided into those that evaluate behaviour, physical health, and physiology.

In aggregated welfare assessments, there have been concerted efforts recently to shift from a reliance on resource-based measures and an evaluation of the presence or absence of welfare harms to increase the proportion of output-based measures and to include both negative and positive welfare indicators [93]. Given the nature of QoL assessments and the animal-centred approach required, indicators that are output-based are recommended [94] and should include unique characteristics and traits of animals as informed by their caregivers, in concert with clinical and physiological evidence [18,59,95,96].

To have confidence in a QoL assessment tool, each indicator and the overall assessment needs to consistently measure what it claims to and produce accurate results over multiple trials. In short, the tool must be both valid and reliable [74,97,98]. In their development of a novel elephant welfare assessment tool, Yon and colleagues [98] demonstrated the importance of evaluating the various types of validity and reliability against predetermined thresholds from the literature. Common types of reliability applied to welfare assessment include inter-/intra-rater, test re-test, and internal consistency. Validity may refer to content, concurrent criterion, or known group criterion validity [98]. Scientific validation of welfare indicators is an active area of research, especially in species such as primates, elephants, and dolphins [74]. To encourage continuous improvement across the taxonomic spectrum, consulting species experts and the existing literature while referencing tools for analogous species is likely to yield the best results [99]. As evident in the development of QoL assessment tools in companion animals, there is a need for more consistency in reporting methodology and statistical validation [20].

#### 3.6.1. Behavioural QoL Indicators

Behavioural observations are often the focus of output-based QoL assessments of captive wild animals [100]. The frequency, duration, and intensity of key behaviours (e.g., vocalization, foraging, aggression, and exploration) may be monitored as a component of, or to complement, species-specific ethograms, time/activity budgets, or space/enclosure usage measures [99,101]. Normal behaviour implies not only natural behaviours, including those that an animal is highly motivated to perform [102], but also an appropriate behavioural range and context for a given individual [103]. For example, locomotor activity may be appropriate if hunting for food but inappropriate in the form of repetitive pacing; likewise, mating may be appropriate if consistent with seasonal patterns but abnormal if excessive.

Where sufficient field research exists, species-typical behaviours in the wild can be used as a baseline for interpretation. Time-budget or behavioural repertoire differences between wild animals and those in captive settings can indicate possible problems with animal management [104], but not all wild behaviours are considered necessary to achieve a good QoL.

Abnormal behaviours, most obviously stereotypies, have been correlated with poor welfare and maladaptation. However, such behaviours may, in some cases, be satisfying or soothing to perform and can be considered a coping mechanism [105]. Other potential indicators of poor welfare or distress may include improper vocalization, extreme timidity, hyper-aggression, excessive sleep, inactivity, escape behaviours, self-mutilation, fur or feather plucking, and decreased performance of behaviours critical to survival and reproduction, but these need to be interpreted in light of the context [106]. Conversely, behaviours that might indicate positive affect include play, affiliative behaviours, approach, allogrooming, and decreased vigilance [99,102]. 

Relying entirely on behavioural data, however, comes with some risk, and a misdiagnosis may have negative consequences [107]. The missteps that can be made in behavioural assessment include mismatches between definitions of animal welfare and the collected data, lack of alternative explanations, faulty logic, behaviour interpreted out of context, murky assumptions, lack of behaviour definitions, and poor justification for assigning a welfare value to a specific behaviour.

Experimentally, more complex behavioural assessments may help define what animals want or need but may be impractical for real-world assessments. A simple choice between alternatives can measure relative preference [108], while motivation can be tested via operant tasks [109]. A preference for something, however, does not necessarily equate to poor QoL in its absence, and animals do not consistently select resources that are in their individual best interests [106]. Additionally, the history and past experiences of animals (e.g., wild-origin versus captive-born) may influence behaviour and coping strategies, akin to the elements of culture and expectation in human QoL literature. 

Recent developments in animal welfare science that warrant further investigation across species as potentially meaningful QoL indicators include measures of cognitive bias to identify positive animal emotions [72,110,111], applications of qualitative behavioural assessments [72], whereby animal body language is examined as a whole rather than as discrete individual behaviours [112], and the use of subjective keeper ratings [113]. Other innovative techniques include social network analyses to better understand social bonds and the associated positive affects [101,114] and quantifying behavioural diversity. High behavioural diversity is inferred to equate to the meeting of animal needs via increased environmental control [115], while low behavioural diversity signals a possible welfare compromise, particularly if associated with stereotypies and/or lethargy [116]. 

#### 3.6.2. Physical Health QoL Indicators

Physical health factors are commonly used QoL indicators that aim to identify deviations from ‘normal’. Measurable physical features include body condition, cleanliness, the presence and severity of lesions and evidence of injury. Physical proxies for behavioural issues can become apparent (e.g., wounds or fur loss from aggressive interactions), but the age and cause of the lesions may be unclear. In some circumstances, direct handling is necessary for measurement, although there is a trend to make use of conditioning programs that facilitate handling-free health evaluations [117].

Veterinary reports can provide retrospective health data for individuals or groups but are reliant on the quality of record-keeping. Longevity is sometimes used as an indirect and crude physical health measure but is insensitive and often controversial [106], especially when referenced in isolation from other parameters.

#### 3.6.3. Physiological QoL Indicators

Physiological indicators have the potential to demonstrate an animal’s response to a situation and are mostly aimed at identifying negative welfare states. Examples include body temperature, respiratory rate, the functioning of the sympathetic–adrenal–medullary axis or the hypothalamic–pituitary–adrenal axis, the levels of other hormones (e.g., prolactin and oxytocin) and measures of immunity (e.g., neutrophil/lymphocyte ratio). Variables such as cost, time, training of personnel and animals, invasiveness, biological fluctuations, social grouping of animals, and handling for sampling or direct measurement determine their real-world utility [61]. Research evaluating the correlation of physiological parameters with welfare has produced mixed results [89], with some measures increasing with stress but also with general arousal, such as during copulation or play [118]. 

Physiological indicators of chronic stress may be particularly significant in assessing the level of everyday welfare of animals in human care. They must be combined with multiple concurrent behavioural observations for context. The relatively recent development of non-invasive techniques (e.g., hormone levels in faeces, urine, or hair) has permitted the incorporation of physiological parameters into QoL assessments of animals, but lack of validation of the tests remains a significant limitation for many zoo species. To illustrate the complexity of validation, in their attempts to monitor adrenocortical activity in 13 species of marsupial, Fanson et al. [119] found that the assay that performed the best varied between even closely related species. 

#### 3.6.4. Pain as a QoL Indicator

Pain, and from a clinical perspective, its effective control, are critically important components of QoL for all sentient species. Attempts to objectively detect and quantify pain should, therefore, be considered an obligatory inclusion in all QoL assessment matrices, particularly HRQoL assessments. When not effectively treated, pain can lead to a damaging effect on all aspects of QoL, including increasing anxiety, aggression, and depressive-like behaviours [120,121,122]. However, pain is a challenging construct to evaluate in animals due to its multi-faceted and subjective nature. Additionally, there are species and individual differences in pain perception and expression.

Measuring acute and chronic pain in animals is often based on carefully interpreted behavioural observations by a veterinarian and/or caregiver [123], evaluating both the presence of abnormal behaviours and the absence of normal behaviours. These findings combined with relevant physical health and physiological parameters (such as locomotor or postural abnormalities, evidence of self-trauma, and elevated heart rate) alongside clinical acumen can assist in the recognition and localization of pain. Determining the underlying cause of pain is the remit of veterinarians and facilitates the development of targeted and effective treatment and management plans. With regard to acute pain, the assessment of facial expressions in the form of Grimace Scales has demonstrated good reliability and validity in domestic mammals [124,125,126], and there is scope to explore their development in mammalian zoo species. Reliable detection and scoring of pain in other vertebrates is challenging, with an acknowledged need to support further research in this area [127,128]. In relation to HRQoL, unless there is evidence to the contrary, one can assume that interventions and conditions that cause pain or distress in humans and domestic species will also cause pain or distress in zoo animals. There are expected to be taxonomic and individual differences in the duration and perceived severity of that pain, the response to analgesics or other treatments, and the resultant impact on QoL. To facilitate the development of evidence-based zoo pain scoring tools, it is crucial to support the ongoing sharing of data between institutions holding the same species.

#### 3.6.5. What Not to Include in a QoL Assessment

In a zoo context, there are many factors that do not influence QoL but that may legitimately influence the decisions that QoL assessments seek to inform, including end-of-life considerations. It is important for transparency that these factors are acknowledged and documented as separate from QoL evaluations and that it is clear how they interact with the results of a QoL assessment. One such factor is the value of an animal’s life, aside from its inherent value as a sentient being. That may be its value to science, to conservation, to the genetic integrity of a species, to educational objectives, to the economic prosperity of a business, or to its cohort. These values may be influenced by culture, politics, emotion, religion, or even the perceived cuteness of the species [52], and there are differing opinions across the international zoo community regarding the value of maintaining post-reproductive animals in spaces that managers could otherwise allocate to breeding or growing animals [81]. Other considerations include the financial, physical resources, or staff time costs of treatment or management changes that might be necessary to improve an animal’s QoL [49]. There may be staff or visitor concern over an animal’s physical appearance even in the absence of any evidence that an abnormality or unusual feature is adversely impacting the animal’s QoL, prompting discussions about the capacity for suitable off-exhibit holding. It may also be relevant to consider the effect of the removal of an animal (via euthanasia or an enclosure or facility move) on the QoL of one or more other animals (e.g., dependent offspring and conspecifics).

For geriatric animals, the terms ‘aged animal assessment’ or ‘euthanasia decision-making tool’ may be used to describe a process that includes a combination of QoL and other considerations. Zoos are encouraged to develop internal processes and documents that clearly delineate the assessment of factors that influence QoL from other considerations. End-of-life decisions made on the basis of QoL are rarely questioned, but it is important that the assessment of QoL relied upon to make such a decision measures QoL from an animal-centric perspective as accurately as possible. Including extraneous factors in a QoL assessment that led to such a decision or referencing only QoL indicators when other considerations were relied upon is disingenuous and could pose a risk to the social license to operate. Trust and transparency are vital for both internal and external zoo stakeholders, particularly surrounding the sensitive topic of euthanasia.

#### 3.6.6. QoL Assessment Challenges

Determining the QoL of zoo animals remains a great challenge for those involved in their management, despite the development of early tools to improve the consistency with which this is done. With the exception of a few large, charismatic mammals (e.g., apes, elephants, dolphins, and some large carnivores), there is a dearth of species-specific guidelines regarding tool design and appraisal. The selection of appropriate measures, establishment of baselines for comparison, analysis of data, interpretation of results, and incorporation of these measures into decision-making and communications remain areas in need of future research [42]. Furthermore, many zoo species are cryptic in nature, and our ability to detect illness, pain, and distress in them is still evolving.

One of the most fundamental difficulties is that, in the absence of validated assessment techniques, many of the indicators that are simple and readily available for incorporation into assessment tools require a professional judgement to be made, and in doing so, a subjective step cannot be avoided. Similarly, the interpretation of the significance of a result in relation to how that will make an animal feel is not completely objective in most circumstances. That subjectivity may be cumulative in a multi-indicator instrument. This inevitable gap in objective deductions about the implications for the animal is often wide enough that people can reach radically different conclusions when assessing an animal’s QoL. In the case of end-of-life decision-making, opinions also often differ regarding the point at which it becomes kinder to euthanize an animal rather than pursue palliative care options [129].

Although purported to make a complex task simpler, the process of designing and interpreting QoL assessments requires the application of the latest sophisticated developments in animal welfare science as well as the incorporation of elements of clinical acumen, knowledge, experience, common sense, and compassion that defy quantification [130].

There is broad agreement on the importance of the concept of QoL as an evaluative criterion [131,132]; however, unlike the formalization and consensus around most biological measurements, the operationalization of QoL remains contested [26,131]. Typically, agreements on measurements stabilize a concept and without this, its utility can be called into question and different ways of operationalizing it can fragment a field [131]. Yet despite the debates for over three decades in human settings, QoL remains an important and essentially unitary concept [131], and there is no suggestion that in animals it is any different.

## 4. Future Directions

### 4.1. Terminology

In the zoo grey literature and general discourse, it is the author’s experience that the term QoL is referenced often, mostly in the context of ‘animal welfare over time’. Animal management examples include describing the effect of appropriate or inappropriate social interactions, enclosure design, environmental enrichment, visitor interactions, or reproductive opportunities. Justifying the decision to undertake an invasive surgical procedure is a veterinary example. In such cases, invariably, there is no attempt to formally quantify QoL, nor is there any description of how a positive or negative effect was determined; it is presumed to be a common sense assumption or inference. Conversely, objective QoL assessments were specifically developed in the zoo field as a precursor to end-of-life management and euthanasia decisions, which has created a situation where some more recent QoL programs intended to be general welfare monitoring tools have been interpreted by staff to mean euthanasia is being considered [133]. This dichotomy in the use of the term ‘QoL’ is not helpful and it can lead to hesitancy in engagement with proposed assessment tools.

Supporting positive animal welfare at all life stages is critical to the mission of accredited zoos and aquariums [4], and it is anticipated that there will be increasing reliance on QoL assessment tools for decision-making in the future. A convergence of the various animal welfare assessment tools in use and development with the QoL assessment construct is suggested to help stabilize the two concepts and avoid fragmenting discussions.

Each institution has the autonomy to decide on the terminology and the specific QoL definition that suits their operation and culture, but as discussed in this review and to avoid confusion, the assessment of animal welfare over time should be considered equivalent to a generic QoL assessment. If, for resourcing and risk-based reasons, QoL assessments are only conducted on geriatric animals, it should be clear that this is a particular form of welfare assessment and the objectives should be clearly outlined. If the assessment focuses entirely on health-related indicators or is designed to evaluate the QoL impacts of a diagnosis, the term HRQoL should be used.

### 4.2. Improving Practice: QoL Assessment Reviews, Refinements, and Research

In the spirit of continuous improvement and to ensure QoL assessment tools are fit for purpose, zoos are encouraged to critically review their current practices regularly and to identify priority areas for refinement. Some suggestions are included here to stimulate initial discussions. It is recommended that any review is preceded by a consideration of how the institution defines QoL, especially as it relates to animal welfare and the animal welfare program in general. Other useful background information includes the existing tools in use, if any, their origins, and staff feedback on the process.

The purpose of QoL assessments in a given zoo (e.g., to inform or seek consensus on euthanasia decision-making, to provide a baseline or benchmark, to predict the impact of one or more diagnoses, to assess proposed individual or species-specific husbandry or management alterations, or to influence the development of care standards) should be made explicit from the outset. A zoo may use QoL assessments for more than one purpose, but it will be important to consider if different tools or adjustments to a generic tool are needed for varied indications. Disease-specific QoL assessments remain largely underutilized in zoo medicine, and a clearer delineation between generic and disease-specific QoL tools and indicators is recommended [9]. Developing optional disease-specific modular components may provide a mechanism to combine these into a single tool where that is deemed appropriate.

To enhance a team’s ability to interpret QoL assessments, the process should begin when: (i) an animal is healthy and behaviourally well, allowing for the development of better individual baselines; and (ii) well in advance of the point at which this information becomes required for end-of-life decision-making [134]. Embracing the exciting and rapidly evolving developments in animal welfare science will undoubtedly create opportunities to incorporate more appropriate indicators. This is especially true for non-mammalian species, where there is a recognized lack of species-specific data for benchmarking and a need to develop evidence-based, reliable and valid welfare metrics. For the high-profile, charismatic species, there are increasingly sophisticated models that can be used to develop similar tools for other taxa. Additionally, the adoption of newer technologies (e.g., bioacoustics, accelerometers, and drones) can help overcome some of the practical limitations of historical QoL assessment techniques [135].

There is ample scope to further develop weighted scales to account for the unique characteristics and indicators that are defined for each species or even, following the trend in human QoL measurement, individuals. Some behaviours, for example, may be a more powerful indicator of QoL than others and, thus, should have greater influence over a QoL assessment or score. The increasing interest in developing individualized measures reflects the understanding that QoL is unique to individuals. While the use of standardized measures and responses selected from a predetermined taxon-specific set is likely to be the most practical approach in zoos, it must be recognized that there is a risk of missing important nuances and developing methods to overcome this are worth considering. 

To better calibrate measures and the cumulative scores of multi-dimensional tools with QoL scaling, there is a need to consider the accumulated knowledge base alongside the following research questions: For a given tool in a particular species, are there clearly defined and defensible score thresholds for positions on a QoL scale? Are the suggested interventions for different positions on the scale evidence-based and appropriate for the species and individual being assessed? Are existing measures and tools appropriate and adequate for all species in a zoo setting, or do new measures need to be developed? Do existing measures and tools take account of changes in adaptation and normalization of previous findings when measuring QoL longitudinally? Are the assessment results correlating well with other data collected in life and with post-mortem findings of euthanized animals? Is it clear what constitutes an important change in QoL to the animal itself?

### 4.3. Embracing the Complexities of Judgement with Science

QoL is a construct that has no physiological basis or specific behavioural signs; instead, it is a composite evaluation of multiple interacting factors, both intrinsic and extrinsic, for a single individual [96]. There are many philosophies about what constitutes a good life and where particular thresholds for intervention lie. By their nature as OROs, animal QoL assessments are imperfect. This imperfection is exacerbated for understudied species, where there is a dearth of baseline behavioural and health data for comparison [90]. These complexities threaten the ambitions of QoL assessments to be truly quantitative and objectivity-promoting. Given that the reliability and validity of many QoL measures currently in use remain undetermined, one may wonder whether there is justification for structured zoo animal QoL assessment tools at all. However, despite their limitations, multi-dimensional QoL assessments are an invaluable instrument for unpacking and documenting complex, ethically challenging animal welfare decisions, for communicating transparently with diverse stakeholders, and for reaching consensus. As such, their early evolution, application, and integration into zoo animal management practices can be viewed as both a science and a professional art. Through sharing experiences, analyses of collated data, and involvement in welfare research, zoos can collaboratively help fill the knowledge gaps necessary to improve the robustness and defensibility of QoL assessments for many species. In doing so, the QoL evaluation tools available will move closer to achieving their intended overarching goal of overcoming the bias-laden approach of simple judgements.

## 5. Conclusions

Quality of life (QoL) is an important and evolving concept for zoo animals that has been converging towards the changing definitions of animal welfare over time. Despite the challenges and limitations in defining and evaluating QoL, it is anticipated that there will be an increase in the need for reliable and valid tools to inform management and care plans in the future. There has been a focus on assessment at the end-of-life stage of large, charismatic mammals to date, but this has been challenged by an ad hoc approach and an absence of baseline benchmarks. Leveraging the development of increasingly robust animal welfare assessment tools in other species at other life stages creates an opportunity for zoo-based QoL evaluations to improve their utility and accuracy. Differentiation of generic and disease-specific assessments, ongoing critical appraisal of assessment design, and acknowledgement of the inherent subjectivity of some aspects of the process are recommended to facilitate the maturation of the QoL assessment process in zoos.

## Figures and Tables

**Table 1:** Selected definitions of quality of life from the human and animal literature.

Definition	Derived from the Human or Animal Literature	Reference
“an individuals’ perception of their position in life in the context of the culture and value systems in which they live and in relation to their goals, expectations, standards and concerns”	Human	[13]
“an overall general well-being that comprises objective descriptors and subjective evaluations of physical, material, social, and emotional well-being together with the extent of personal development and purposeful activity, all weighted by a personal set of values”	Human	[14]
“a conscious cognitive judgment of satisfaction with one’s life”	Human	[12]
“the subjective and dynamic evaluation by the individual of its circumstances (internal and external) and the extent to which these meet its expectations (that may be innate or learned and that may or may not include anticipation of future events), which results in, or includes, an affective (emotional) response to those circumstances (the evaluation may be a conscious or an unconscious process, with a complexity appropriate to the cognitive capacity of the individual)”	Animal	[1]
“a continuum from a life not worth living (with poor welfare and suffering) through an adequate life (a life worth living with minimal suffering) to a good life (with good welfare and mainly positive emotions and experiences)”	Animal	[23]
“the affective (emotional) response of an individual to his or her circumstances, and the extent to which the circumstances meet his or her expectations”	Animal	[24]
“an individual’s satisfaction with its physical and psychological health, its physical and social environment and its ability to interact with that environment”	Animal	[16]
“a matter of how valuable each animal’s life is for that animal”	Animal	[25]

## Data Availability

No new data were created or analyzed in this study. Data sharing is not applicable to this article.

## References

[1] Scott E.M., Nolan A.M., Reid J., Wiseman-Orr M.L. (2007). Can we really measure animal quality of life? Methodologies for measuring quality of life in people and other animals. Anim. Welf..

[2] Garratt A., Schmidt L., MacIntosh A., Fitzpatrick R. (2002). Quality of life measurement: Bibliographic study of patient assessed health outcome measures. BMJ.

[3] Pierce J., Shanan A., Shanan A., Pierce J., Shearer T. (2017). Quality of life in the animal hospice and palliative care patient. Hospice and Palliative Care for Companion Animals: Principles and Practice.

[4] Krebs B.L., Marrin D., Phelps A., Krol L., Watters J.V. (2018). Managing aged animals in zoos to promote positive welfare: A review and future directions. Animals.

[5] Harley J., Clark F.E. (2019). Animal Welfare Toolkit.

[6] Bolechova P., Clauss M., de Man D., Galeffi C., Hofman S., Kappelhof J., Kfir G., Kjellson B., Kölpin T., Lawrenz A., European Association of Zoos and Aquaria (2020). EAZA Best Practice Guidelines for Elephants.

[7] Association of Zoos and Aquariums Animal Welfare Program Template. https://assets.speakcdn.com/assets/2332/welfare_program_template_final.pdf.

[8] Moons P., Budts W., De Geest S. (2006). Critique on the conceptualisation of quality of life: A review and evaluation of different conceptual approaches. Int. J. Nurs. Stud..

[9] Karimi M., Brazier J. (2016). Health, health-related quality of life, and quality of life: What is the difference?. PharmacoEconomics.

[10] Prutkin J.M., Feinstein A.R. (2002). Quality-of-life measurements: Origin and pathogenesis. Yale J. Biol. Med..

[11] Peasgood T., Brazier J., Mukuria C., Rowen D. (2014). A Conceptual Comparison of Well-Being Measures Used in the UK.

[12] Rijeski W.J., Mihalko S.L. (2001). Physical activity and quality of life in older adults. J. Gerontol. Ser. A Biol. Sci. Med. Sci..

[13] Whoqol Group (1995). The World Health Organization quality of life assessment (WHOQOL): Position paper from the World Health Organization. Soc. Sci. Med..

[14] Felce D., Perry J. (1995). Quality of life: Its definition and measurement. Res. Dev. Disabil..

[15] Cummins R.A. (2005). Moving from the quality of life concept to a theory. J Intellect Disabil Res..

[16] Belshaw Z., Asher L., Harvey N.D., Dean R.S. (2015). Quality of life assessment in domestic dogs: An evidence-based rapid review. Vet. J..

[17] Broom D.M. (2007). Quality of life means welfare: How is it related to other concepts and assessed. Anim. Welf..

[18] Yeates J., Main D.C.J. (2009). Assessment of companion animal quality of life in veterinary practice and research. J. Small Anim. Pract..

[19] Arndt S.S., Goerlich V.C., van der Staay F.J. (2022). A dynamic concept of animal welfare: The role of appetitive and adverse internal and external factors and the animal’s ability to adapt to them. Front. Anim. Sci..

[20] Fulmer A.E., Laven L.J., Hill K.E. (2022). Quality of life measurement in dogs and cats: A scoping review of generic tools. Animals.

[21] Long M., Dürnberger C., Jenner F., Kelemen Z., Auer U., Grimm H. (2022). Quality of life within horse welfare assessment tools: Informing decisions for chronically ill and geriatric horses. Animals.

[22] Lawrence A.B., Vigors B., Sandøe P. (2019). What is so positive about positive animal welfare?—A critical review of the literature. Animals.

[23] FAWC (2009). Farm Animal Welfare in Great Britain Past Present and Future.

[24] Reid J., Wiseman-Orr M.L., Scott E.M., Nolan A.M., Gaynor J., Muir W. (2015). Health related quality of life measurement. Handbook of Veterinary Pain Management.

[25] Yeates J. (2016). Quality of life and animal behaviour. Appl. Anim. Behav. Sci..

[26] Post M.W. (2014). Definitions of quality of life: What has happened and how to move on. Top. Spinal Cord Inj. Rehabil..

[27] Kaplan R.M., Bush J.W. (1982). Health-related quality of life measurement for evaluation research and policy analysis. Health Psychol..

[28] Spitzer W.O. (1987). State of science 1986: Quality of life and functional status as target variables for research. J. Chronic Dis..

[29] Shiovitz-Ezra S., Leitsch S., Graber J., Karraker A. (2009). Quality of life and psychological health indicators in the national social life, health, and aging project. J. Gerontol. B Psychol. Sci. Soc. Sci..

[30] Wemelsfelder F. (2007). How animals communicate quality of life: The qualitative assessment of animal behaviour. Anim. Welf..

[31] van Leeuwen K.M., van Loon M.S., van Nes F.A., Bosmans J.E., de Vet H.C.W., Ket J.C.F., Widdershoven G.A.M., Ostelo R.W.J.G. (2019). What does quality of life mean to older adults? A thematic synthesis. PLoS ONE.

[32] McMillan F.D. (2003). Maximizing quality of life in ill animals. J. Am. Anim. Hosp. Assoc..

[33] Green T.C., Mellor D.J. (2011). Extending ideas about animal welfare assessment to include ‘quality of life’ and related concepts. N. Z. Vet. J..

[34] Mellor D.J., Beausoleil N.J., Littlewood K.E., McLean A.N., McGreevy P.D., Jones B., Wilkins C. (2020). The 2020 five domains model: Including human–animal interactions in assessments of animal welfare. Animals.

[35] Cussen V.A., DiGangi B.A., DiGangi B.A., Cussen V.A., Reid P.J., Collins K.A. (2022). Welfare and ethical decision-making. Animal Behavior for Shelter Veterinarians and Staff.

[36] Honess P., Wolfensohn S. (2010). The Extended Welfare Assessment Grid: A matrix for the assessment of welfare and cumulative suffering in experimental animals. Altern. Lab. Anim..

[37] McMillan F.D. (2000). Quality of life in animals. J.-Am. Vet. Med. Assoc..

[38] Belshaw Z. (2018). Quality of life assessment in companion animals: What, why, who, when and how. Companion Anim..

[39] Pennacchini M., Bertolaso M., Elvira M.M., De Marinis M.G. (2011). A brief history of the Quality of Life: Its use in medicine and in philosophy. Clin. Ter..

[40] Megari K. (2013). Quality of life in chronic disease patients. Health Psychol. Res..

[41] Innes A., Surr L. (2001). Measuring the well-being of people with dementia living in formal care settings: The use of Dementia Care mapping. Aging Ment. Health.

[42] Higginson I.J., Carr A.J. (2001). Measuring quality of life: Using quality of life measures in the clinical setting. BMJ.

[43] Martin A.J., Stockler M. (1998). Quality-of-life assessment in health care research and practice. Eval. Health Prof..

[44] Fayers P.M., Hopwood P., Harvey A., Girling D.J., Machin D., Stephens R. (1997). Quality of life assessment in clinical trials: Guidelines and a checklist for protocol writers. The UK Medical Research Council experience. Eur. J. Cancer.

[45] Asada Y. (2005). Assessment of the health of Americans: The average health-related quality of life and its inequality across individuals and groups. Popul. Health Metr..

[46] Solans-Domenech M., Pane S., Estrada M.-D., Serra-Sutton V., Berra S., Herdman M., Alonso J., Rajmil L. (2008). Health-related quality of life measurement in children and adolescents: A systematic review of generic and disease-specific instruments. Value Health.

[47] Mullan S. (2015). Assessment of quality of life in veterinary practice: Developing tools for companion animal carers and veterinarians. Vet. Med..

[48] Tidière M., Gaillard J.M., Berger V., Müller D.W., Lackey L.B., Gimene O., Clauss M., Lemaître J.F. (2016). Comparative analyses of longevity and senescence reveal variable survival benefits of living in zoos across mammals. Sci. Rep..

[49] Föllmi J., Steiger A., Walzer C., Robert N., Geissbühler U., Doherr M.G., Wenker C. (2007). A scoring system to evaluate physical condition and quality of life in geriatric zoo mammals. Anim. Welf..

[50] Vogelnest L., Talbot J.J., Miller R.E., Lamberski N., Calle P. (2019). Quality-of-life assessment and end-of-life planning for geriatric zoo animals. Fowler’s Zoo and Wild Animal Medicine Current Therapy.

[51] Vinette Herrin K., Vogelnest L., Hulst F., Tobias G., Campbell-Ward M., Bryant B., Pitcher B. Evaluation of an aged animal assessment tool used for quality-of-life assessment and end-of-life planning for geriatric zoo animals. Proceedings of the American Association of Zoo Veterinarians Conference.

[52] Wolfensohn S. (2020). Too cute to kill? The need for objective measurements of quality of life. Animals.

[53] Quain A., Ward M.P., Mullan S. (2021). Ethical challenges posed by advanced veterinary care in companion animal veterinary practice. Animals.

[54] Persson K., Selter F., Neitzke G., Kunzmann P. (2020). Philosophy of a “Good Death” in small animals and consequences for euthanasia in animal law and veterinary practice. Animals.

[55] Shanan A. (2011). A veterinarian’s role in helping pet owners with decision making. Vet. Clin. Small Anim. Pract..

[56] Selter F., Persson K., Kunzmann P., Neitzke G. (2023). End-of-life decisions: A focus group study with German health professionals from human and veterinary medicine. Front. Vet. Sci..

[57] Mather J.A. (2019). Ethics and care: For animals, not just mammals. Animals.

[58] Nolan E.C., Neiffer D., Terrell S., Dias J., Miller A., Piltz J., Odell K., Gonio M., Peccie G., Christman J. Geriatric animal care and end-of-life decision-making in zoological institutions. Proceedings of the American Association of Zoo Veterinarians Conference.

[59] Lynch S., Savary-Bataille K., Leeuw B., Argyle D.J. (2011). Development of a questionnaire assessing health-related quality-of-life in dogs and cats with cancer. Vet. Comp. Oncol..

[60] Sherwen S.L., Hemsworth L.M., Beausoleil N.J., Embury A., Mellor D.J. (2018). An animal welfare risk assessment process for zoos. Animals.

[61] Wolfensohn S., Shotton J., Bowley H., Davies S., Thompson S., Justice W.S.M. (2018). Assessment of welfare in zoos: Towards optimum quality of life. Animals.

[62] Kiddie J., Collins L. (2015). Identifying environmental and management factors that may be associated with the quality of life of kennelled dogs (*Canis familiaris*). Appl. Ani. Behav. Sci..

[63] Riley L.M., Rose P.E. (2020). Concepts, applications, uses and evaluation of environmental enrichment: Perceptions of zoo professionals. J. Zoo Aquar. Res..

[64] Riggio G., Pirrone F., Lunghini E., Gazzano A., Mariti C. (2020). Zookeepers’ perception of zoo canid welfare and its effect on job satisfaction, worldwide. Animals.

[65] Abresch R.T., Carter G.T., Han J.J., McDonald C.M. (2009). New clinical end points in rehabilitation medicine: Tools for measuring quality of life. Am. J. Hosp. Palliat. Med..

[66] Reid J., Wiseman-Orr M.L., Scott E.M., Nolan A. (2013). Development, validation and reliability of a web-based questionnaire to measure health-related quality of life in dogs. J. Small Anim. Prac..

[67] United States Food and Drug Administration (2009). Patient-Reported Outcome Measures: Use in Medical Product Development to Support Labeling Claims.

[68] McCusker J., Stoddard A.M. (1984). Use of a surrogate for the Sickness Impact Profile. Med. Care.

[69] Haverman L., Limperg P.F., Young N.L., Grootenhuis M.A., Klaassen R.J. (2017). Paediatric health-related quality of life: What is it and why should we measure it?. Arch. Dis. Child..

[70] Mariti C., Gazzano A., Moore J.L., Baragli P., Chelli L., Sighieri C. (2012). Perception of dogs’ stress by their owners. J. Vet. Behav..

[71] Niessen S.J.M. (2011). Quality-of-life assessment: Honouring our oath in practice and research. J. Small Anim. Pract..

[72] Browning H. (2022). Assessing measures of animal welfare. Biol. Philos..

[73] Coleman G. (2018). Public animal welfare discussions and outlooks in Australia. Anim. Front..

[74] Jones N., Sherwen S.L., Robbins R., McLelland D.J., Whittaker A.L. (2022). Welfare assessment tools in zoos: From theory to practice. Vet. Sci..

[75] Giuffrida M.A., Kerrigan S.M. (2014). Quality of life measurement in prospective studies of cancer treatments in dogs and cats. J. Vet. Intern. Med..

[76] Doit H., Dean R.S., Duz M., Brennan M.L. (2021). A systematic review of the quality of life assessment tools for cats in the published literature. Vet. J..

[77] Haralstad K., Wahl A., Andenæs R., Andersen J.R., Andersen M.H., Beisland E., Borge C.R., Engebretsen E., Eisermann M., Halvorsrud L. (2019). A systematic review of quality of life research in medicine and health sciences. Qual. Life Res..

[78] Connolly M.A., Johnson J.A. (1999). Measuring quality of life in paediatric patients. Pharmacoeconomics.

[79] Eiser C., Morse R. (2001). Quality-of-life measures in chronic diseases of childhood. Health Technol. Assess..

[80] Church M.L., Priehs D.R., Denis H., Croft L., DiRocco S., Davis M. (2018). Technique, postoperative complications, and visual outcomes of phacoemulsification cataract surgery in 21 penguins (27 eyes): 2011–2015. Vet. Ophthalmol..

[81] Watters J., Sulzner K., Marrin D., Huang S., MacDonald C., Ostapak S., Poole A., Hayle H. (2015). Assessing quality of life in geriatric zoo animals. WAZA Mag..

[82] Lambeth S.P., Schapiro S.J., Bernacky B.J., Wilkerson G.K. (2013). Establishing ‘quality of life’ parameters using behavioural guidelines for humane euthanasia of captive non-human primates. Anim. Welf..

[83] Hosey G., Melfi V., Pankhurst S. (2009). Zoo Animals: Behaviour, Management and Welfare.

[84] Hill S.P., Broom D.M. (2009). Measuring zoo animal welfare: Theory and practice. Zoo Biol..

[85] Main D.C.J., Webster A.J.F., Green L.E. (2001). Animal welfare assessment in farm assurance schemes. Acta Agric. Scand. A Anim. Sci..

[86] Cohen S., Ho C. (2023). Review of rat (*Rattus norvegicus*), mouse (*Mus musculus*), guinea pig (*Cavia porcellus*), and rabbit (*Oryctolagus cuniculus*) indicators for welfare assessment. Animals.

[87] Benn A.L., McLelland D.J., Whittaker A.L. (2019). A review of welfare assessment methods in reptiles, and preliminary application of the Welfare Quality^®^ protocol to the pygmy blue-tongue skink, *Tiliqua adelaidensis*, using animal-based measures. Animals.

[88] Shepherdson D., Carlstead K., Wielebnowski N. (2004). Cross-institutional assessment of stress responses in zoo animals using longitudinal monitoring of faecal corticoids and behaviour. Anim. Welf..

[89] Maple T.L., Perdue B.M. (2013). Zoo Animal Welfare.

[90] O’Brien S.L., Cronin K.A. (2023). Doing better for understudied species: Evaluation and improvement of a species-general animal welfare assessment tool for zoos. Appl. Anim. Behav. Sci..

[91] Yeates J.W., Main D.C.J. (2008). Assessment of positive welfare: A review. Vet. J..

[92] Gaalema D.E., Perdue B.M., Kelling A.S. (2011). Food preference, keeper ratings, and reinforcer effectiveness in exotic animals: The value of systematic testing. J. Appl. Anim. Welf. Sci..

[93] Richmond S.E., Wemelsfelder F., de Heredia I.B., Ruiz R., Canali E., Dwyer C.M. (2017). Evaluation of animal-based indicators to be used in a welfare assessment protocol for sheep. Front. Vet. Sci..

[94] Whitham J.C., Wielebnowski N. (2013). New directions for zoo animal welfare science. Appl. Anim. Behav. Sci..

[95] Villalobos A.E. Quality of Life Scale Helps Make Final Call. Veterinary Practice News, September 2004. https://www.veterinarypracticenews.com/quality-of-life-scale-helps-make-final-call/.

[96] Oyama M.A., Rush J.E., O’Sullivan M.L., Williams R.M., Rozanski E.A., Petrie J.P., Sleeper M.M., Brown D.C. (2008). Perceptions and priorities of owners of dogs with heart disease regarding quality versus quantity of life for their pets. J. Am. Vet. Med. Assoc..

[97] Del Greco L., Walop W., McCarthy R.H. (1987). Questionnaire development: 2. Validity and reliability. Can. Med. Assoc. J..

[98] Yon L., Williams E., Harvey N.D., Asher L. (2019). Development of a behavioural welfare assessment tool for routine use with captive elephants. PLoS ONE.

[99] Manteca Vilanova X. EAZA Animal Welfare Webinars: The Fundamentals of Animal Welfare Assessments, 2020. https://www.youtube.com/watch?v=8TT6xBLcggU.

[100] Watters J.V., Margulis S.W., Atsalis S. (2009). Behavioral monitoring in zoos and aquariums: A tool for guiding husbandry and directing research. Zoo Biol..

[101] Rose P.E., Riley L.M. (2021). Conducting behavioural research in the zoo: A guide to ten important methods, concepts and theories. J. Zool. Bot. Gard..

[102] McPhee M.E., Carlstead K., Kleiman D.G., Thompson K.V., Baer C.K. (2010). The importance of maintaining natural behaviors in captive mammals. Wild Mammals in Captivity: Principles and Techniques for Zoo Management.

[103] Bacon H. (2018). Behaviour-based husbandry—A holistic approach to the management of abnormal repetitive behaviours. Animals.

[104] Melfi V.A., Feistner A.T.C. (2002). A comparison of the activity budgets of wild and captive Sulawesi crested black macaques (*Macaca nigra*). Anim. Welf..

[105] Mason G.J. (1991). Stereotypies: A critical review. Anim. Behav..

[106] Kagan R., Veasey J., Kleiman D.G., Thompson K.V., Baer C.K. (2010). Challenges of zoo animal welfare. Wild Mammals in Captivity: Principles and Techniques for Zoo Management.

[107] Watters J.V., Krebs B.L., Eschmann C.L. (2021). Assessing animal welfare with behavior: Onward with caution. J. Zoo. Bot. Gard..

[108] Fraser D., Phillips P.A., Thompson B.K. (1993). Environmental preference testing to access the well-being of animals: An evolving paradigm. J. Agric. Env. Ethics.

[109] Kirkden R.D., Pajor E.A. (2006). Using preference, motivation and aversion tests to ask scientific questions about animals’ feelings. Appl. Anim. Behav. Sci..

[110] Brydges N.M., Leach M., Nicol K., Wright R., Bateson M. (2011). Environmental enrichment induces optimistic cognitive bias in rats. Anim. Behav..

[111] Clegg I.L.K., Rödel H.G., Mercera B., van der Heul S., Schrijvers T., de Laender P., Gojceta R., Zimmitti M., Verhoeven E., Burger J. (2019). Dolphins’ willingness to participate (WtP) in positive reinforcement training as a potential welfare indicator, where WtP predicts early changes in health status. Front. Psychol..

[112] Fleming P.A., Clarke T., Wickham S.L., Stockman C.A., Barnes A.L., Collins T., Miller D.W. (2015). The contribution of qualitative behavioural assessment to appraisal of livestock welfare. Anim. Prod. Sci..

[113] Whitham J.C., Wielebnowski N. (2009). Animal-based welfare monitoring: Using keeper ratings as an assessment tool. Zoo Biol..

[114] Rose P., Croft D.P. (2015). The potential of Social Network Analysis as a tool for the management of zoo animals. Anim. Welf..

[115] Asher L., Collins L.M., Ortiz-Pelaez A., Drewe J.A., Nicol C.J., Pfeiffer D.U. (2009). Recent advances in the analysis of behavioural organization and interpretation as indicators of animal welfare. J. R. Soc. Interface.

[116] Miller L.J., Vicino G.A., Sheftel J., Lauderdale L.K. (2020). Behavioral diversity as a potential indicator of positive animal welfare. Animals.

[117] Hellmuth H., Augustine L., Watkins B., Hope K. (2012). Using operant conditioning and desensitization to facilitate veterinary care with captive reptiles. Vet. Clin. N. Am. Exot. Anim. Pract..

[118] Coria-Avila G.A., Pfaus J.G., Orihuela A., Domínguez-Oliva A., José-Pérez N., Hernández L.A., Mota-Rojas D. (2022). The neurobiology of behavior and its applicability for animal welfare: A review. Animals.

[119] Fanson K.V., Best E.C., Bunce A., Fanson B.G., Hogan L.A., Keeley T., Narayan E.J., Palme R., Parrott M.L., Sharp T.M. (2017). One size does not fit all: Monitoring faecal glucocorticoid metabolites in marsupials. Gen. Comp. Endocr..

[120] Parent A.J., Beaudet N., Beaudry H., Bergeron J., Bérubé P., Drolet G., Sarret P., Gendron L. (2012). Increased anxiety-like behaviors in rats experiencing chronic inflammatory pain. Behav. Brain Res..

[121] Belshaw Z., Yeates J. (2018). Assessment of quality of life and chronic pain in dogs. Vet. J..

[122] Ferreira-Chamorro P., Redondo A., Riego G., Leánez S., Pol O. (2018). Sulforaphane inhibited the nociceptive responses, anxiety and depressive-like behaviors associated with neuropathic pain and improved the anti-allodynic effects of morphine in mice. Front. Pharmacol..

[123] Reid J., Nolan A.M., Scott E.M. (2018). Measuring pain in dogs and cats using structured behavioural observation. Vet. J..

[124] Dalla Costa E., Minero M., Lebelt D., Stucke D., Canali E., Leach M.C. (2014). Development of the Horse Grimace Scale (HGS) as a pain assessment tool in horses undergoing routine castration. PLoS ONE.

[125] Evangelista M.C., Watanabe R., Leung V.S.Y., Monteiro B.P., O’Toole E., Pang D.S.J., Steagall P.V. (2019). Facial expressions of pain in cats: The development and validation of a Feline Grimace Scale. Sci. Rep..

[126] Mota-Rojas D., Olmos-Hernández A., Verduzco-Mendoza A., Hernández E., Martínez-Burnes J., Whittaker A.L. (2020). The utility of grimace scales for practical pain assessment in laboratory animals. Animals.

[127] La’Toya V.L. (2023). Pain recognition in reptiles. Vet. Clin. N. Am. Exot. Anim. Pract..

[128] Mikoni N.A., Sanchez-Migallon Guzman D., Paul-Murphy J. (2023). Pain recognition and assessment in birds. Vet. Clin. N. Am. Exot. Anim. Pract..

[129] Kirkwood J.K. (2007). Quality of life: The heart of the matter. Anim. Welf..

[130] Kagan R., Carter S., Allard S. (2015). A universal animal welfare framework for zoos. J. Appl. Anim. Welf. Sci..

[131] Armstrong D., Caldwell D. (2004). Origins of the concept of quality of life in health care: A rhetorical solution to a political problem. Soc. Theory Health.

[132] Fox M.W. (2019). Determining animals quality of life: Veterinary criteria and assessment. Arch. Vet. Sci. Med..

[133] Evans B. Zoo Quality of Life Program: Refinements Needed to Set Clear Expectations. Metro: Portland, USA, 2017. https://www.oregonmetro.gov/sites/default/files/2017/11/20/Zoo%20quality%20of%20life%20program%20audit.pdf.

[134] Simpson L., Grove J., Bronson E., Herrelko E.S. (2023). Quality of life assessment: The value of longitudinal data in making the end-of-life care decision for a macaque (*Macaca Silenus/Macaca Nemestrina*). J. Appl. Anim. Welf. Sci..

[135] Whitham J.C., Miller L.J. (2016). Using technology to monitor and improve zoo animal welfare. Anim. Welf..

